# Reproductive Resilience to Food Shortage in a Small Heterothermic Primate

**DOI:** 10.1371/journal.pone.0041477

**Published:** 2012-07-25

**Authors:** Cindy I. Canale, Elise Huchard, Martine Perret, Pierre-Yves Henry

**Affiliations:** 1 UMR 7179 CNRS-MNHN, Département Ecologie et Gestion de la Biodiversité, Muséum National d’Histoire Naturelle, Brunoy, France; 2 Behavioural Ecology and Sociobiology Unit, German Primate Center GmbH (DPZ), Göttingen, Germany; 3 Courant Research Centre “Evolution of Social Behaviour,” Georg-August University, Göttingen, Germany; Institut Pluridisciplinaire Hubert Curien, France

## Abstract

The massive energetic costs entailed by reproduction in most mammalian females may increase the vulnerability of reproductive success to food shortage. Unexpected events of unfavorable climatic conditions are expected to rise in frequency and intensity as climate changes. The extent to which physiological flexibility allows organisms to maintain reproductive output constant despite energetic bottlenecks has been poorly investigated. In mammals, reproductive resilience is predicted to be maximal during early stages of reproduction, due to the moderate energetic costs of ovulation and gestation relative to lactation. We experimentally tested the consequences of chronic-moderate and short-acute food shortages on the reproductive output of a small seasonally breeding primate, the grey mouse lemur (*Microcebus murinus*) under thermo-neutral conditions. These two food treatments were respectively designed to simulate the energetic constraints imposed by a lean year (40% caloric restriction over eight months) or by a sudden, severe climatic event occurring shortly before reproduction (80% caloric restriction over a month). Grey mouse lemurs evolved under the harsh, unpredictable climate of the dry forest of Madagascar and should thus display great potential for physiological adjustments to energetic bottlenecks. We assessed the resilience of the early stages of reproduction (mating success, fertility, and gestation) to these contrasted food treatments, and on the later stages (lactation and offspring growth) in response to the chronic food shortage only. Food deprived mouse lemurs managed to maintain constant most reproductive parameters, including oestrus timing, estrogenization level at oestrus, mating success, litter size, and litter mass as well as their overall number of surviving offspring at weaning. However, offspring growth was delayed in food restricted mothers. These results suggest that heterothermic, fattening-prone mammals display important reproductive resilience to energetic bottlenecks. More generally, species living in variable and unpredictable habitats may have evolved a flexible reproductive physiology that helps buffer environmental fluctuations.

## Introduction

A fundamental assumption in life-history theory is that reproduction is costly [1]. For most small mammals, energy requirements generally increase throughout the reproductive cycle [2]–[4]. Prior to fecundation, the energetic cost of reproductive effort (gametogenesis, courtship, mating) is probably sex-dependent and possibly high [5], but overall poorly quantified [6]. Gestation and lactation are critical periods of energy expenditure [7]. During gestation, foetal growth and the development of reproductive organs (uterus, placenta, mammary), entail a drastic increase of energy expenditure. After birth, lactation is even more costly [4], [8]–[11]. For instance, in the guinea pig (*Cavia porcellus*), the daily energy expenditure during gestation is 2.4 times the basal metabolic rate (BMR) and 3.7 times the BMR during lactation [12]. The extent of such costs place reproductive decisions at the core of life-history trade-offs [13], [14].

In seasonal environments, breeding phenology has been selected so that the costliest reproductive stages match the annual peak in food availability [15]–[17]. Some organisms even use the same proximate cues to time both the energy balance and reproduction [18]–[20]. However, new concerns arise from climate change since it generates phenological mismatches between the energetic needs of animal reproduction and environmental productivity [17], [21], [22]. Primary and secondary producers seem to track changes in the timing and intensity of climatic variations with relative accuracy; in contrast, consumers, particularly those with a slow reproductive rate, would be less flexible [23]–[26]. Reproductive resilience - defined as the ability to maintain a constant reproductive output despite unexpected environmental disturbances – is an alternative response that received much less attention than phenological adjustments [27], [28]. Understanding whether, and to which extent, females can flexibly adjust their energetic investment to reproduction according to unpredicted food shortages is essential to predict whether organisms could compensate climate changes by plastic phenotypic adjustments [22], [28], [29].

Physiological studies documented various effects of food restriction on female reproductive output. In small laboratory mammals, it can inhibit mating behavior [30]–[33] and delay sexual maturation, potentially resulting in acyclicity or anoestrus [34], [35]. Gestation can be shortened and even compromised by a higher risk of miscarriage and fetal death, while fetal growth can be reduced. Eventually, energetic deficits can affect juvenile growth, behavior and survival [36], [37]. Despite all these potentially deleterious effects of food shortage, skipping a reproductive event can be a risky decision. This is particularly true for seasonal breeders with fast life histories because survival to the next cycle is uncertain, while an entire lifetime offers, at best, a handful of reproductive opportunities [38]. Thus, it might still prove safer to prioritize immediate over future reproduction, at least if a flexible reproductive physiology allows it.

Species that evolved in poorly predictable, harsh environments are good candidates to document reproductive resilience to unexpected food shortages [27], [39]. Our model is a small heterothermic primate, the grey mouse lemur (*Microcebus murinus*), which evolved under the unpredictable climatic conditions of the dry forests of Madagascar [40]. There, the alternation of seasons is typically predictable, with abundant food during the wet season, and drastic food shortage during the dry season. But the high interannual climatic variability, connected to El Niño and the Indian Ocean Dipole oscillations [40] makes the suitability of the wet season and the harshness of the dry season poorly predictable, essentially through important between-year variations in the abundance and temporal distribution of rainfall. Organisms with a wide range of energy saving mechanisms were selected by food shortage caused by both the seasonality, and recurrent climatic anomalies [40]. These include daily torpor (periodic hypometabolism; [41], [42]) and energy storage in adipose tissues. Torpor use is flexibly adjusted to energetic constraints such as caloric restriction [43]–[45], and can be used by reproductive females [46]. However, it remains unknown whether, and to which extent, such mechanisms might allow maintaining reproductive output under energetic shortage.

Mouse lemurs display relatively fast life-history. They reach sexual maturity within their first year, breed seasonally once a year [47], [48], and reproduce on average twice in their life [49]. In the wild, they mate at the end of the dry season when food availability is minimal and when fat stores are depleted, meaning that female grey mouse lemurs have probably evolved to cope with limited energy supply before the mating season [1], [48]. Given this life-history, we expected that female mouse lemurs would prioritize immediate over future reproduction in situations of food shortage, and would maintain energetic allocation to reproduction, potentially at the expense of their own survival.

To quantify the extent to which female reproductive effort and output is adjusted to unusual, unfavorable conditions, we exposed captive female mouse lemurs, maintained at thermoneutrality [52], either to food available *ad libitum* (control), to a chronic food shortage (40% food restriction over eight months) or to an acute food shortage (80% food restriction over a month). The moderate, long-term restriction was designed to simulate a relatively bad year due to below-average primary and secondary production, with long-lasting effects over a full reproductive cycle [50]. The acute, short-term restriction was designed to simulate a harsh climatic event occurring shortly before the onset of the reproductive season [28]. The dependency of female reproductive performances on energetic availability was assessed at two crucial stages: (i) during ovulation and gestation - the least energetically costly stages, and (ii) during lactation - the most expensive stage. We predicted that (i) early stages would be the most resilient to food shortage. In contrast, (ii) during lactation, when the energetic demand peaks and the ontogenetic program of offspring development is fully launched, the physiological adjustments may not suffice to compensate food shortages, and such energetic deficit should occur at greater expense for the females (in terms of body mass loss). Hence, offspring growth is expected to be less resilient to food shortage than the initial reproductive effort.

## Materials and Methods

### Ethics Statement

All experiments were performed in accordance with the European Communities Council Directive (86/609/EEC). The research was conducted at authorized facilities (#91–305) by authorized experimenters (#91–439 & #91–455, issued by the Departmental Veterinary Service of *Essonne*, France), and protocols had been approved by the internal review board of UMR 7179. The levels of calorie restriction that were used in experiments do not induce chronic physiological stress [43], [51]. At the end of the experiments, all animals were returned to standard living conditions.

### Animals and Housing Conditions

Grey mouse lemurs were obtained from our laboratory breeding colony (Brunoy, *Museum National d’Histoire Naturelle*, France, European Institution Agreement No. 962773). The animals used in experiments were sexually mature and multiparous females, aged between 2 and 4 years. All animals are maintained in mono-sexual groups (apart for mating days) under standard breeding conditions at thermo-neutral ambient temperature (24–25°C; [52]) and a relative humidity of 55%. Experiments were implemented at thermoneutrality to remove the potentially confounding effects of energetic costs related to thermoregulation, and to ensure comparability across studies. Seasonal variations of physiological functions are artificially maintained by alternating a 6-month period of summer-like long days (14 h of artificial light per day), with a 6-month period of winter-like short days (10 h of artificial light per day) [53]. Animals were fed with a standardized homemade mixture of spice bread, egg, concentrated milk, white cheese, baby cereals and mixed fresh banana blended all together with water [44]. The macronutrient composition of the mixture is 50% carbohydrates, 20% proteins, and 30% lipids, with a caloric value of 4.8 kJ.g^−1^. Water was available *ad libitum*.

### Experimental Designs

Two experiments were conducted to measure adjustments of the reproductive investment in response to chronic (Experiment 1) and acute (Experiment 2) food shortage. Experiment 1 consisted in twelve control females fed *ad libitum* (AL) and 12 females facing a 40% chronic caloric restriction (CR60; i.e., their daily food supply represented 60% of the food mass offered to AL females). This moderate, long-term caloric restriction lasted over eight months, from the beginning of the previous winter season to the weaning of juveniles. In Experiment 2, 36 females were divided into two groups: 18 control females were fed *ad libitum* (AL) and 18 females were exposed to an 80% acute caloric restriction (CR20; i.e., their daily food supply represented 20% of the food mass offered to AL females). This acute, short-term restriction lasted 35.5±0.8 days. It started three weeks before the transition to long days, and was maintained until the first day of oestrus. This acute food shortage was not extended to the subsequent, more energetically demanding, stages of reproduction since it was judged potentially too invasive with respect to animal welfare.

To control individual food intake, all females were housed individually in cages (50×50×50 cm) with branches and two nest-boxes. Following the transition to long days, female reproductive state was monitored daily. Females were given the opportunity to mate by housing them with three males the first day of their vaginal opening (indicating oestrus). Female grey mouse lemurs are sexually receptive during a short time window lasting few hours around oestrus day, meaning that all copulations occur during one single night per year [48], [54]. During mating nights, food was provided *ad libitum* but sexual harassment by males largely prevented females from feeding [5]. Non-mated females were returned to their individual cage when vagina resealed, on average 2.6±0.5 days after oestrus (maximum: 3 days).

### Reproductive Traits

The influence of both chronic (Experiment 1) and acute (Experiment 2) food shortages was tested on the early stages of reproduction (mating success, fertility), whereas, for ethical reasons (cf. here before), only the effect of chronic food shortage (Experiment 1) was tested on the later stages of reproduction (gestation, reproductive output, lactation, and offspring growth).

#### Mating success and fertility (Experiments 1–2)

The timing of reproduction was characterized by the date of oestrus, defined as the number of days separating the onset of the long day period from the day of vaginal opening. Mating success was determined by checking for the presence (mating success) or absence (no mating) of a vaginal plug and/or sperm in the vaginal tract [54].

Fertility was assessed by estradiol variations at oestrus, measured in urine samples (0.25–1 ml) collected on the day of oestrus 1–1.5 hour before night, and stored at −80°C. Since they were collected by spontaneous urination during handling, urine samples were only obtained for a subset of females (n = 26 AL, n = 9 CR60 and n = 15 CR20). Urinary 17ß-Estradiol (later referred to as urinary E2) concentrations were measured on 25 µl of urine in duplicates using an enzyme-linked immunosorbent assay (DE- 2693, Demeditec, Germany). Percentages of cross-reactivity are: Estradiol-17ß 100%, estrone 0.2%, estriol 0.05%. The mean intra- and inter-assay coefficients of variation are 4.7 and 7.8% respectively. The minimum detectable level in urine was 9.7 pg.mL^−1^. To control for variations in diuresis, the concentration of creatinine of each sample was measured with an enzyme immunoassay (8009, Metra®Creatinine, Quidel Corporation, San Diego, USA). Urinary E2 values were expressed in pg of E2 per mg of Creatinine (pg.mg^−1^.Cr [49]).

#### Gestation and reproductive output (Experiment 1)

Maternal allocation to fetal growth was related to gestation length and litter characteristics. Pregnant females were weighed (±0.1 g) every 15 days from the day of oestrus to 60 days post-oestrus. Pregnant females were monitored daily around the expected date of parturition. Gestation length was defined as the difference between copulation date and parturition date. Fetal growth was calculated by dividing the litter mass at birth by gestation length. Gestational effort was calculated as (total litter body mass at birth/maternal body mass at oestrus)*100. Litter size is defined as the number of pups per female at birth. Litter sex-ratio was determined at birth. Reproductive success was defined as the number of pups weaned and measured 45 days after birth [55].

#### Lactation and offspring growth (Experiment 1)

Maternal energetic investment in offspring growth was quantified through body mass variations of the mother and pups from the day of parturition until day 45. Lactating mothers were weighed every five days. Offspring femur length was measured with a numerical caliper (±0.01 mm, three measures) 5 days after birth and then every 5 days during 45 days. Body mass was taken (±0.1 g) on the same dates, to measure body condition growth (i.e., body mass growth after adjusting for body size growth). ‘AL pups’ and ‘CR pups’ were named after the food supply of their mother (*ad libitum* and CR60, respectively).

### Data Analyses

Data are reported as mean ± standard error of the mean (SEM). All statistical analyses were implemented with R ver. 2.8.1 [56]. Non-parametric tests were applied for non-binary data when sample sizes were less than 15 observations.

#### Standard statistical analyses

Urinary concentration of estradiol (after log-transformation), female body mass at oestrus, gestation length and gestational effort were analyzed with linear models. The date of oestrus was analyzed with GLMs fitted with a Poisson distribution and a log-link function. Mating success was fitted with a binomial distribution and a logit-link function. In case of overdispersion, quasi-distribution models were used. Body masses of gestating, lactating females and of pups were analyzed with linear mixed models (LMM). The random effects were female identity to account for the non-independence among repeated measures when analyzing maternal body mass, and litter identity to account for the non-independence of offspring within litters when analyzing pup body mass. These LMMs were built with the ‘lme’ function from the ‘nlme’ package. Model fits were adjusted for non-independencies of residual data points due to heteroscedasticity among treatments or to temporal autocorrelation [57].

**Table 1:** Effects of food availability on female condition (body mass), fertility (timing of oestrus, urinary estradiol level at oestrus - noted urinary E2) and mating success.

	Reproductive parameter (response variable)			
Fixed effect	Body mass	Mating success	Date of oestrus	Urinary E2
	[g, N = 60]	[logit(%), N = 59]	[log(day), N = 59]	[log(pg.mg^−1^Cr.), N = 52]
Experiment	3.83±8.19	2.21±0.96	−0.42±0.14	1.64±0.30
	F_1,57_ = 0.77, p = 0.32	χ^2^_1_ = 4.93, **p = 0.03**	F_1,57_ = 9.00, **p<0.01**	F_1,51_ = 71.91, **p<10^−3^**
Food	−25.76±8.73	0.34±0.82	−0.20±0.14	0.27±0.33
	F_1,56_ = 39.46, **p<10^−3^**	χ^2^_1_ = 0.59, p = 0.44	F_1,56_ = 0.41, p = 0.52	F_1,50_ = 3.82, p = 0.07
Food × Experiment	−14.13±11.20	−1.59±1.21	0.26±0.20	0.19±0.41
	F_1,55_ = 1.60, p = 0.21	χ^2^_1_ = 1.80, p = 0.18	F_1,55_ = 1.84, p = 0.18	F_1,49_ = 0.22, p = 0.64

‘Experiment’ accounts for potential differences in baseline levels among experiments 1 and 2. ‘Food effect’ accounts for the effect of food shortage (CR individuals, whatever the level of food shortage) *versus* no food limitation (AL individuals). ‘Food × Experiment’ interaction accounts for a difference in the impact of chronic *versus* acute caloric restriction (see methods for more details). Estimates (mean ±SE) and tests were obtained from linear models for body mass and E2 (after log-transformation), and with generalized linear models with a log-link (date of oestrus) or a logit-link function (Mating success). Significant differences are indicated in bold. Urinary E2 values were expressed in pg of E2 per mg of Creatinine.

**Table 2:** Reproductive parameters of females fed *ad libitum* (AL) or exposed to either a 40% chronic caloric restriction (CR60) or a 80% acute caloric restriction (CR20).

	Experiment 1		Experiment 2	
	AL	CR60	AL	CR20
**Female fertility and mating success**				
Body mass at oestrus (g)	119.6±13.6	83.3±4.6	113.9±7.3	74.0±2.8
Female mating success (%)	41.7	50	86.7	65
Date of oestrus (days)	18.1±1.2	14.8±1.5	11.9±1.2	12.7±1.1
Urinary E2 (pg.mg^−1^ Cr)	149±28.9	174.3±21.9	864.3±181.8	1207.8±175.9
**Gestation and reproductive output**				
Gestation length (days)	62.6±0.8	62.5±0.9	–	–
Litter body mass at birth (g)	17.9±3.3	12.9±1.9	–	–
Fetal growth (g.day^−1^)	0.28±0.05	0.21±0.03	–	–
Maternal body mass at oestrus (g)	119.6±13.6	83.3±4.6	–	–
Gestational effort (%)	12.7±1.8	14.6±1.9	–	–
Litter size (nb pups at birth)	3.0±0.4	2.3±0.4	–	–
Litter sex ratio at birth (males per females)	1.4±0.5	0.4±0.8	–	–
Reproductive success (nb weaned pups)	2.4±0.4	2±0.4	–	–

Fixed effects were tested with likelihood-ratio tests (or F-tests in case of overdispersion) among nested models, by deletion from the final model containing only significant effects. Date of oestrus, urinary E2 and mating success were analyzed using models containing data from both Experiments 1 and 2. To adjust data for potential differences in baseline levels among experiments (i.e., different means for AL individuals), we included the effect of Experiment identity (1 or 2) as a fixed effect in the analysis. Then, the test of the ‘Food effect’ accounted for the effect of food shortage (CR individuals, whatever the level of food shortage) *versus* no food limitation (AL individuals). In this analytical design, a difference in the impact of chronic *versus* acute caloric restriction was tested through the presence of an interaction between the Experiment and the Food effects in our model. For later reproductive stages (Experiment 1 only), we tested for the fixed effect of food availability only. To analyze how females allocate energy to gestation and lactation, we adjusted female body mass, gestation length and gestational effort for the additive, linear effect of litter size. Sampling date (‘Days effect’) was also introduced as a fixed effect to model the linear temporal variation in the response variable, and inter-individual variability in this temporal response was accounted for by including a random slope parameter for the Days effect [58]. All interactions among simple effects (Food, Litter size, Days) were included in the initial models.

#### Growth and body condition analyses

Growth parameters for offspring were estimated with 2-parameter Gompertz growth curves ([59]; fitted with the ‘nlme’ function of package ‘nlme’ [60]) of the form:
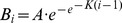
where *B_i_* was the dimension measured (bone length or body mass) at age *i*, *A* is the asymptotic value, and *K* is the daily exponential growth rate. The model also included the fixed effects of the food treatment on *A* and/or *K*, and a random term for among-individual variation.

**Figure 1:**
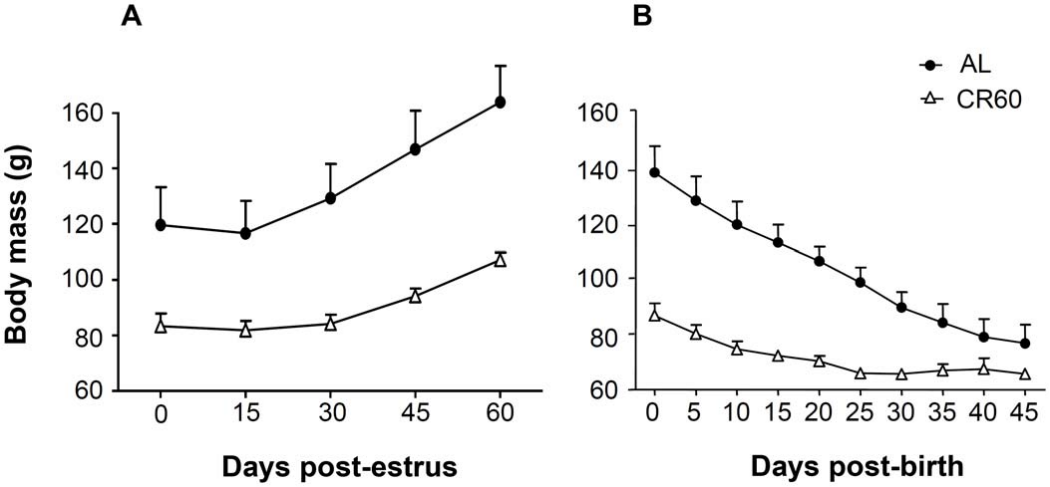
Effect of food availability on body mass over (A) gestation and (B) lactation. Body mass (±SEM) of females fed *ad libitum* (AL) and calorie restricted females (CR60) (A) during the two months following oestrus, and (B) during the 45 days after birth.

**Table 3:** Effects of food availability (Food effect: females fed *ad libitum* vs. females exposed to a chronic food restriction), time of gestation (Days effect; numbered from the day of copulation), and their interaction, on body mass of (a) gestating and (b) lactating females (N = 11 females from Experiment 1).

Fixed effect	Estimate	Test
**(a)Gestation**		
Days	0,78±0.07	F_1,43_ = 122.3, **p<10** ^−**3**^
Food	−40.70±11.90	F_1,9_ = 13.4, **p<10** ^−**2**^
Food × Days	−0.40±0.09	F_1,42_ = 20.4, **p<10** ^−**3**^
**(a)Lactation**		
Days	−0.91±0.16	F_1,108_ = 124.5, **p<10** ^−**3**^
Food	−35.67±8.56	F_1,9_ = 33.4, **p<10** ^−**3**^
Litter size	9.01±1.68	F_1,108_ = 39.1, **p<10** ^−**3**^
Days × Food	0.74±0.19	F_1,105_ = 4.1, **p = 0.04**
Days × Litter size	−0.32±0.07	F_1,105_ = 4.7, **p = 0.03**
Food × Litter size	4.92±3.15	F_1,105_ = 1.4, p = 0.23
Days × Food × Litter size	0.07±0.15	F_1,104_ = 0.0, p = 0.96

For lactation, we also accounted for the effect of litter size. Estimates (mean ±SE) and tests were obtained from linear mixed models. Significant differences are in bold.

**Table 4:** Effect of food availability to mothers (Food effect: females fed *ad libitum* or exposed to a 40% chronic caloric restriction) and of litter size on gestation length, fetal growth and gestational effort (N = 11 females from Experiment 1).

	Reproductive	parameter (response	variable)
Fixed effect	Gestation length	Fetal growth	Gestational effort
	(days)	(g.day^−1^)	(%)
Food	−0.24±1.41	−0.02±0.02	4.73±1.08
	F_1,8_ = 0.03, p = 0.87	F_1,8_ = 0.81, p = 0.39	F_1,8_ = 19.06, **p<10** ^−**3**^
Litter size	−0.21±0.72	0.08±0.01	4.09±0.55
	F_1,8_ = 0.06, p = 0.80	F_1,8_ = 68.65, **p<10** ^−**3**^	F_1,8_ = 39.87, **p<10** ^−**3**^
Food × Litter size	−3.00±1.05	−0.03±0.02	0.89±1.14
	F_1,7_ = 8.13, **p = 0.02**	F_1,7_ = 2.65, p = 0.15	F_1,7_ = 0.61, p = 0.46

Estimates (mean ±SE) and tests were obtained from linear mixed models. Significant differences are indicated in bold.

Departure from a simple Gompertz curve would indicate that the food treatment altered the regularity of growth. To test this, we fitted a Gompertz model to femur length data for each food treatment, and extracted the residuals. If the Gompertz growth curve satisfactorily described the variation in body size with age, residual size should not be related to age. If the relationship is significantly non-linear, then growth actually differed from simple, exponential growth, and irregularities in the pace of growth were identified graphically (i.e., the predicted value for a given age differed by more than 2 S.E from 0). This was done with Generalized Additive Mixed Models (GAMMs; one per food treatment) of the form: Residual Size ∼ s(Age), where the term *s* (a spline function) is a smoothing parameter for the effect of age. If the number of degrees of freedom of *s* significantly differs from 1, then the relationship is non-linear [60]. GAMMs were fitted with the ‘gamm’ function of the ‘mgcv’ package.

**Figure 2:**
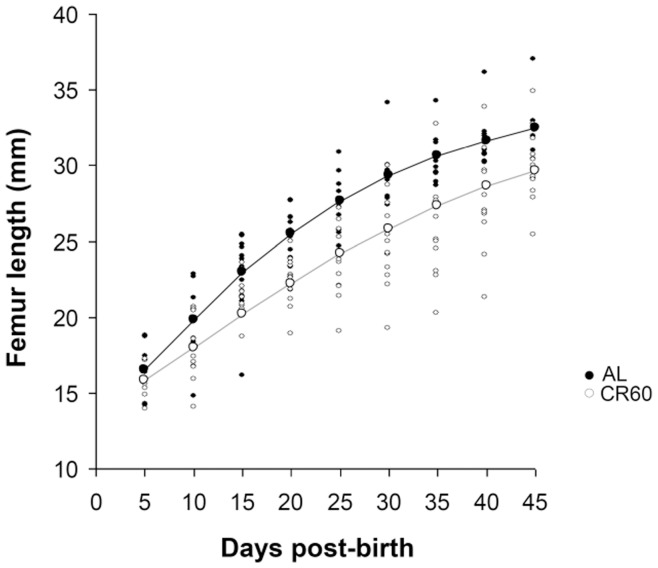
Effect of food availability to lactating mothers on offspring femur growth. Femur length grew slower and less for pups reared by calorie restricted females (CR60, open circles, grey line) than for those reared by females fed *ad libitum* (AL, black dots, black line). Lines draw the growth curves estimated per treatment with 2-parameters Gompertz models.

The transition between lactation and weaning is expected to have a greater influence on the trajectory of offspring body condition for CR offspring than for AL offspring (at least in captivity where food is provided *ad libitum* to juveniles), since weaned offspring do not depend anymore on their mother to acquire food. To test for such a ‘catch-up’ effect of weaning on body condition, we proceeded as follow. To distinguish whether body mass gain was due to size growth *versus* to an improving body condition (i.e., the growing organs and fat stores), we analyzed non-linear variations of body mass adjusted for body size (indexed by femur length) with age [61]. This was done by fitting a GAMM separately for each food treatment, with the form: Log(Body mass) ∼ Log(Femur) + *s*(Age). The spline function for the age effect tested for the existence of a non-linear variation in body condition with age. The tipping-points in the growth of body condition were identified graphically.

**Figure 3:**
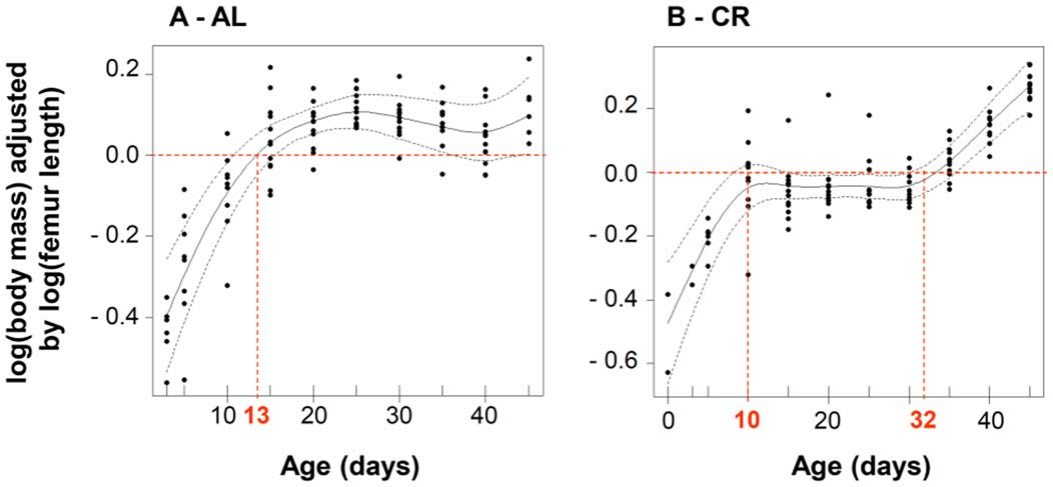
Effect of food availability to lactating mothers on the variation of offspring body condition with age. Non-linear temporal variation of log (body mass) residuals [adjusted for log (femur length)] of offspring from females fed *ad libitum* (AL, panel A) and from calorie restricted females (CR, panel B). The curve describing the age effect (solid line) was extracted from GAMMs. Dashed lines depict 2 standard error point-wise confidence bands, and black dots provide partial residuals. In panel A., red dashed line points the date at which body condition is above average, thereby signing a sustained high body mass increase relative to body size growth. In panel B., red dashed lines and bold labels delimit the period of delayed growth.

## Results

### Female Fertility and Mating Success

Food restriction effectively reduced the body mass at oestrus (effect Food, Table 1), with a comparable magnitude in both experiments (Table 2). However, it did not affect female mating success (63%), the date of oestrus (13.9±0.7 days) and the urinary concentration of estradiol at oestrus (719.6±103.3 pg.mg^−1^ Cr., Table 1). The summary statistics for these parameters are given in Table 2. The interaction between food availability and experiment was not significant for any of the reproductive parameters examined, suggesting that females respond in similar ways when they face brief and acute *versus* chronic and moderate food shortage.

### Gestation and Reproductive Success

The temporal variation of body mass was influenced by food treatment (effect Days × Food; Table 3A, Fig. 1A). AL females gained weight faster than CR60 females over the course of gestation (1.1±0.06 and 0.4±0.07 g.day^−1^, respectively). The adjustment of gestation length to litter size depended on food availability: when food was not limiting, gestation length was longer when there were more pups (+1.5±0.6 days.pup^−1^ in AL females), whereas the relationship was in the opposite direction when females faced a chronic food shortage (−1.5±0.8 days.pup^−1^ in CR60 females; effect Food × Litter size, Table 4, Fig. S1). Fetal growth did not differ according to food treatment (0.24±0.02 g.day^−1^, Table 4; see Table 4 for more details on litter body mass at birth). Relative gestational effort increased with litter size (+4.1±0.5%.pup^−1^, effect Litter size, Table 4, Fig. S2) and was significantly influenced by food availability (Table 4): at birth, AL and CR60 litter mass represented 12.7±1.8% and 14.6±1.9% of the post-partum mass of females respectively (Table 2).

Litter size (2.6±0.3 pups) did not depend on food availability, suggesting an absence of adjustment of litter size to food availability (H = 0.93, df = 1, p = 0.30). Litter sex-ratio tended to differ between food treatments (χ^2^_1_ = 3.18, p = 0.07) with CR60 having more females than males (AL: 1.4±0.5 males per female; CR60: 0.4±0.8).

### Lactation and Offspring Growth

During lactation, daily loss in maternal body mass was greater for AL females (−1.7±0.2 g.day^−1^) than for CR60 females (−1.1±0.1 g.day^−1^; effect Days × Food, Table 3B, Fig. 1B). The number of pups weaned (2.2±0.3) was not adjusted according to food availability (H = 0.61, df = 1, p = 0.43).

Offspring body size increased with age following a classical 2-parameters Gompertz function (Fig. 2). Perinatally food-restricted pups grew slower than those whose mother was fed *ad libitum*: the exponential growth rate (*K*) was 0.058±0.005 for control pups and 0.035±0.003 for CR pups (F_1,181_ = 11.4, p = 0.004, Fig. 2). The asymptotic femur length was 34.7±1.0 mm for AL pups and 36.7±3.4 mm for CR pups (F_1,181_ = 36.18, p<0.001). In offspring raised by mothers fed *ad libitum*, body size growth was satisfactorily described by the Gompertz curve, as there was no relationship linking the residuals of femur length to age (spline function, F_1,100_ = 0.23, p = 0.63, Fig. S3A). In contrast, the non-linear dependence of residual femur length on age in CR animals (spline function, F_3.7,100.3_ = 3.9, p<0.001) revealed a delayed growth between 25 and 34 days of age (Fig. S3B), and a return to a normal growth trajectory afterwards.

Body mass increased with body size, but the relationship linking both variables differed between treatments (effect log(femur) × Food, F_1,182_ = 5.47, p = 0.02), with body condition of AL pups gaining 1.71±0.07 g per mm of femur growth, whereas CR pups gained only 1.47±0.06 g.mm^−1^. The increase of body condition (i.e., of body mass adjusted for femur growth) with age was non-linear (significant spline functions for AL pups, F_5,95_ = 16.25, p<0.001, and for CR pups, F_6.2,97.8_ = 15.48, p<0.001). Body condition increased slower than femur length until the age of 10–13 days. Afterward, the positive residuals indicate that AL individuals maintained a constant, high body mass increase relative to body size growth (Fig. 3A). CR juveniles followed a markedly different trajectory: their body mass increased linearly with body size until 32 days of age, after which they suddenly initiated a sustained increase in body condition (Fig. 3B). Since weaning usually takes place around the age of 45 days (Fig. 3A; [55]), this indicated an early weaning in CR offspring.

## Discussion

Our study highlights the reproductive resilience of a small, heterothermic primate to experimentally-induced unpredictable food shortages simulating those induced by ‘bad’ years or extreme climatic events (summarized in Table 5). As predicted, the early stages of reproduction (from ovulation to birth) appeared to be the most resilient to food reduction. Food-deprived females, even under intense calorie restriction, ovulated, mated [5], and became pregnant at similar rates as control females. Their estrogenization was independent of food availability (and is also independent of body mass, [62]). They did not delay nor skip a reproductive cycle, which is congruent with the fact that reproductive timing is under strong photoperiodic control [47], [63], [64]. During gestation, mothers prioritized resource allocation to fetuses over their own body condition: they maintained birth litter size and mass constant despite gaining less mass than control females. During the later stages of reproduction (from birth to weaning), food-deprived females fell short of energy and could not ensure a normal offspring growth, which suggests that their physiological capacity to optimize energy allocation was not unlimited. Delayed pup growth before weaning probably resulted from reduced milk production in food restricted females, which is supported by their lower daily loss in body mass compared to control females. This lower resilience of reproductive performances to food shortage fits the distribution of energetic costs in mammalian reproduction, which peaks during lactation [1], [11]. Furthermore, the chronology of the female reproductive cycle implies that females might exhaust their fat reserves prior to lactation. Taken together, our results show that food deprived females managed to maintain one of the most significant fitness components, i.e. their number of surviving offspring at weaning. We are aware that the acceptance of null hypotheses (*i.e.* no effect of a treatment) is not a formal demonstration of the absence of an effect, i.e. reproductive resilience. Nonetheless, the accumulation of non-significant tests on independent variables (Table 1) still conveys information on the globally weak effect of caloric restriction on reproduction.

**Table 5:** Summary table of the effects of food shortage on female reproductive parameters in the present study.

Reproductive stage	Variable	Type of adjustment	Statistical impact of food shortage	Table or Figure
Early	**Date of oestrus**	Timing of allocation	**None**	Table 1
Exp. 1 - AL/CR60, Exp. 2 - AL/CR20	**Urinary estradiol levels at oestrus**	Allocation	**None**	Table 1
	**Mating success**	Allocation	**None**	Table 1
Early	Gestation length	Timing of allocation	Decreased for large litters	Table 3, Fig. S1
Exp. 1 - AL/CR60	**Fetal growth**	Allocation	**None**	Table 3
	**Gestational effort**	Allocation	**Increased**	Table 3, Fig. S2
	**Litter size at birth**	Allocation	**None**	_
	**Litter sex-ratio at birth**	Allocation	**None** (but a trend for more females)	_
Late	Offspring size growth rate	Allocation	Decreased and delayed	Fig. 2, S3
Exp. 1 - AL/CR60	**Offspring asymptotic size**	Allocation	**Increased**	Fig. 2
	**Offspring body condition growth**	Allocation	Delayed, with **catch-up growth**	Fig. 3
	Date of weaning	Timing of allocation	Advanced	Fig. 3
	**Reproductive success (Nb of** **weaned pups)**	Allocation	**None**	_

The non-detection – or positive – statistical effects of food shortage (in bold) are considered as indicative of resilience of the allocation to reproduction to food shortage. ‘Exp.’ holds for Experiment.

### The Physiological Bases of Reproductive Resilience

Our results stand in contrast with virtually all previous experimental work (also conducted at thermoneutrality) on the impact of food shortage on the reproduction of small mammals (see Introduction). This difference is likely explained by compensatory physiological strategies. First, mouse lemurs have an exceptional propensity to store fat over prolonged periods to overcome the long dry season [65]. Fat deposits accumulated during the non-reproductive period could serve as an additional energy supply gradually depleted over the breeding season, as hares do [66]. The gradual shift from a predominantly ‘capital’ (where females rely on their body fat stores) to an ‘income’ (where females rely on their daily food intake) strategy over the breeding season [67], should be faster in food restricted females as demonstrated by their rapid drop in body mass (see also [68]). Second, food restriction enhances the expression of energy saving mechanisms, particularly the reduction of thermoregulatory costs through torpor use [43], [44], [69]. In non-reproductive mouse lemurs, a 40% food restriction induces an increase by 7 to 8 hours of torpor duration [43], [44], [69]. Torpor has been observed in pregnant and lactating females of various species [70]–[74], including mouse lemurs [46]. It allows them to re-allocate energy typically spent into basal metabolic costs to reproductive costs, with a flexible schedule adjusted to environmental variations [75]. The energetic efficiency of torpor use may be further enhanced by social thermoregulation: when related, reproductive females aggregate in the same nests [76], [77] as in reproductive bats [78]. The physiological responses should be different in free-ranging mouse lemurs due to competing energetic demands between reproduction, foraging and thermoregulation. It would thus now be interesting to complement our experimental results with an investigation of the extent of phenotypic plasticity in reproductive traits of wild females across a gradient of environmental productivity [78].

### The Ecological Significance of Reproductive Resilience

Considering the ecology of mouse lemurs further helps to understand why they evolved this capacity to compensate food shortages during early reproductive stages. Female mouse lemurs invest their ultimate energetic reserves to mate and become pregnant at the end of the dry season, 3.5 months before the annual peak in food availability [4], [47], [48], [79]. Hence female physiology may have been selected to allow mating and gestating despite minimal energy availability. In contrast, lactation typically takes place after the first rains when food availability is expected to be maximal [4], [77], [80]–[82]. Yet, the risk of falling short of energy during the late phase of their reproduction is non-negligible, because the temporal fluctuations and quality of food are still prone to strong inter-annual climatic variations, for instance in El Niño years [40], [83]. Consequently, the ability to launch physiological mechanisms of energetic compensation during lactation may also have been selected for.

Increasing gestational effort to maintain litter size under uncertain foraging conditions might appear like a risky decision. But delaying the first (and often unique: [48]) reproductive event of the year would cause a shift of offspring weaning time, or of the second births. In both cases, offspring might not be able to grow and build-up fat stores to face their first dry season. Furthermore, relying on the chance of having better, future reproductive options would be particularly risky given that a female reaching maturity has a short life expectancy (2.3 years: [49]), and that favorable conditions are poorly predictable [40]. Hence, females would better grasp any immediate chance of reproducing, whatever the environmental conditions are, possibly counting on future rains to enhance offspring condition and refill their own stores (e.g., [82]).

### The Extent and Limits of Reproductive Resilience

The resilience of the female reproductive output may not fully buffer against food shortage. Several direct and indirect costs may still compromise the inclusive fitness of food deprived females. First, by reallocating their own energy stores towards offspring, females might compromise their own survival prospect or future fecundity [84]. Second, food shortage delayed offspring growth, because females failed to maintain their energetic balance, or because some of the compensatory physiological mechanisms, such as torpor, had negative side-effects on nursing or milk supply [72], [85]–[88]. Delayed growth can impact offspring fitness in several ways. First, it can impair survival to the first dry season. Second, it can lower reproductive performances during the first mating season [89]–[91]. Third, there is increasing evidence that early maternal environment, by contributing in shaping adult phenotypes, can alter lifetime performances and fitness [92]. Energetic limitation during ontogeny could alter offspring cognitive performances (although, in our experiments, these impairments became undetectable by the age of 10 months with *ad libitum* feeding; [93]). Fourth, the offspring of food deprived mothers underwent a catch-up growth after weaning (as in [94]). Although such a compensatory growth likely allows the recovery of normal chances of survival, it may still bear significant lifetime costs through an accelerated senescence [95]. Eventually, food shortage could even reduce the inclusive fitness of females. Mouse lemur females are polyandrous, and mating with multiple partners might allow them to obtain high quality sperm, and possibly good genes for their offspring [96]. However, food shortage would prevent access to these benefits because only females in good condition are polyandrous [5]. Overall, the relative importance of these costs and the extent to which they limit the adaptive potential of reproductive resilience to food shortage remains to be established.

### Conclusion

Organisms that evolved under unpredictable, drastically seasonal environments might have developed flexible reproductive strategies to cope with predictable intra-annual, as well as less predictable inter-annual, food shortages. Captive mouse lemurs, under thermo-neutral conditions, maintain a high gestational effort despite food shortage to maintain birth timing and litter size, at the cost of depleting their own reserves and weaning lighter offspring. We propose that reproductive resilience can be interpreted into a coherent ecological framework, following the evolution of energy saving mechanisms and predictions generated by life-history theory. Progress in understanding the eco-physiological rules driving reproductive decisions in response to environmental unpredictability now requires gathering experimental data from organisms with diverse life-histories exposed to a range of energetic constraints (thermoregulatory and foraging costs). There is a real need to exploit field observations to document the responses of heterotherms to natural, unpredicted energetic bottlenecks, for instance by taking advantage of climatic accidents.

## Supporting Information

Figure S1 **Effects of food availability and litter size on gestation length.** AL holds for females fed *ad libitum* (plain lines), and CR60 for calorie restricted females (dashed lines). Lines illustrate the predicted values of the final linear model controlling for experiment identity.
(TIF)

Figure S2 **Effect of litter size on gestational effort.** AL holds for females fed *ad libitum*, and CR60 for calorie restricted females. The line represents the predicted values of a linear model controlling for experiment identity.
(TIF)

Figure S3 **Effect of food availability to lactating mothers on the regularity of offspring femur growth.** Temporal variation of femur length residuals (i.e., adjusted for Gompertz growth) of offspring from females fed *ad libitum* (AL, panel A.) and calorie restricted females (CR, panel B.). A value of 0 for the spline term indicates average residual femur length. The spline curve describing the smoothed effect of age (solid line) was estimated using a GAMM. Dashed lines depict 2 standard error point-wise confidence bands, and black dots provide partial residuals. In panel B., red dashed lines and bold labels delimit the period of delayed growth.
(TIF)
