# Supplementary figures and images for: Reproductive Resilience to Food Shortage in a Small Heterothermic Primate
Source: PLoS One. 2012 Jul 25;7(7):e41477. doi: 10.1371/journal.pone.0041477 (PMC3405090; doi:10.1371/journal.pone.0041477)

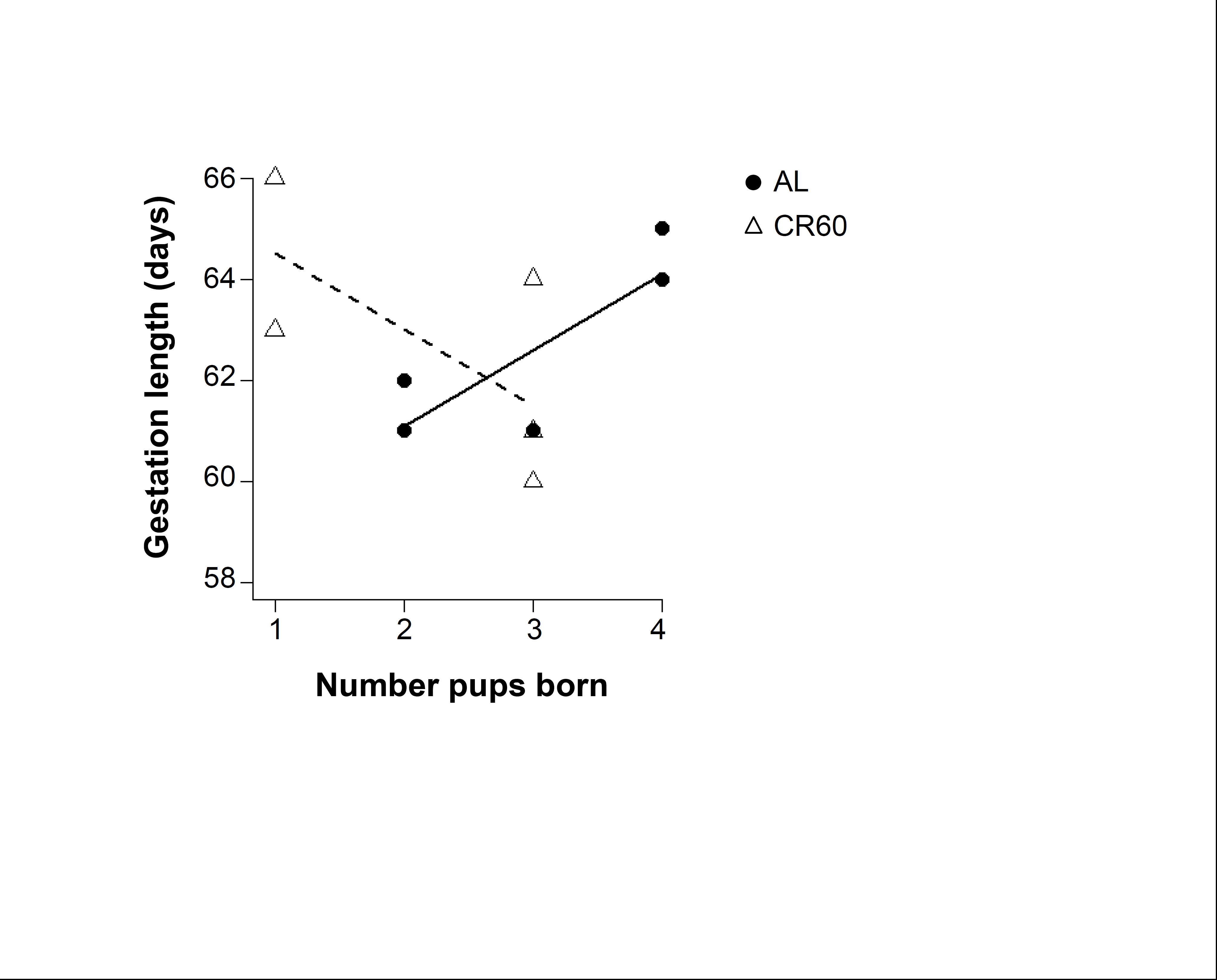

Supplement: Figure S1 — Effects of food availability and litter size on gestation length. AL holds for females fed ad libitum (plain lines), and CR60 for calorie restricted females (dashed lines). Lines illustrate the predicted values of the final linear model controlling for experiment identity. (TIF) [file pone.0041477.s001.tif]

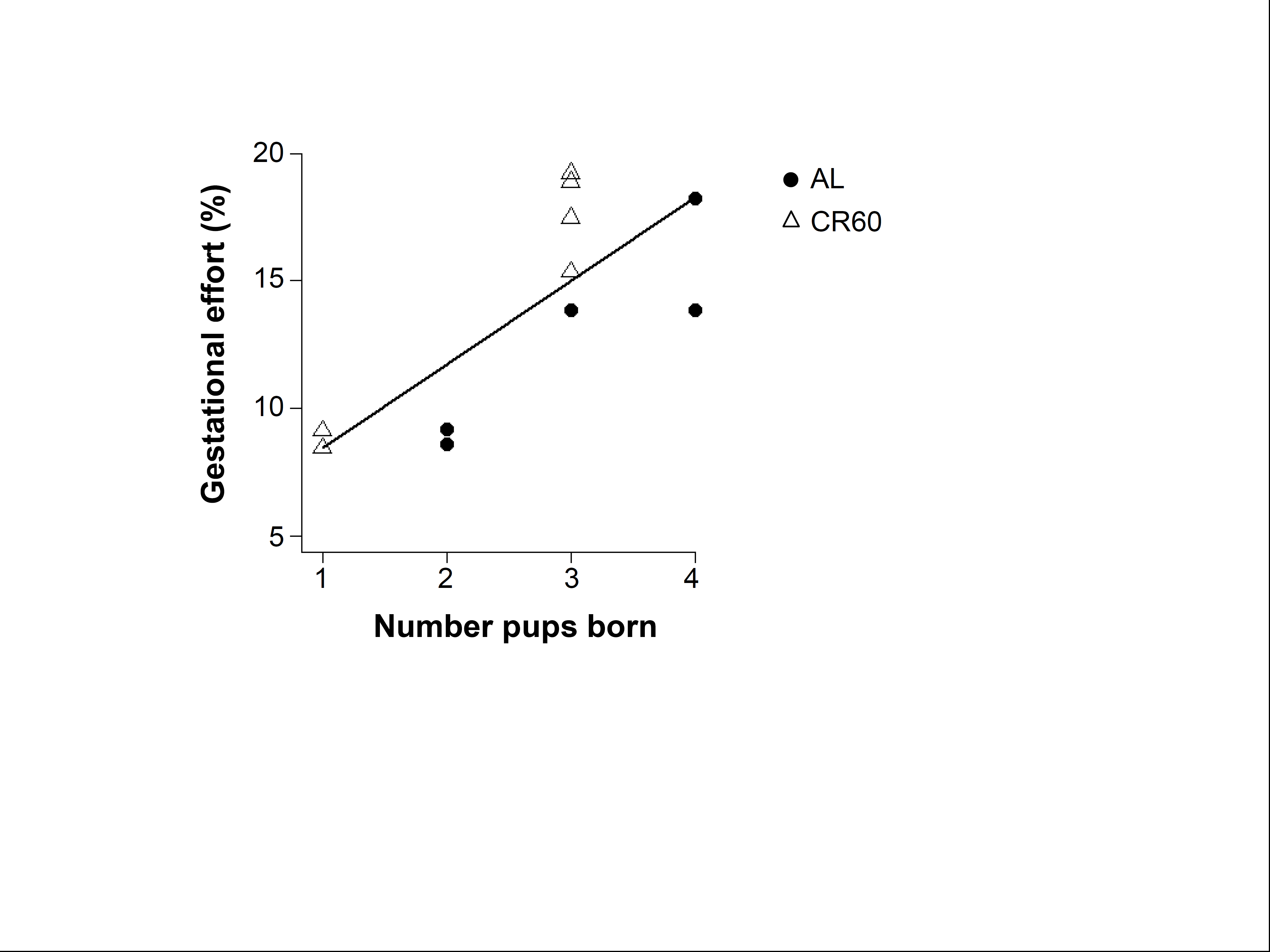

Supplement: Figure S2 — Effect of litter size on gestational effort. AL holds for females fed ad libitum, and CR60 for calorie restricted females. The line represents the predicted values of a linear model controlling for experiment identity. (TIF) [file pone.0041477.s002.tif]

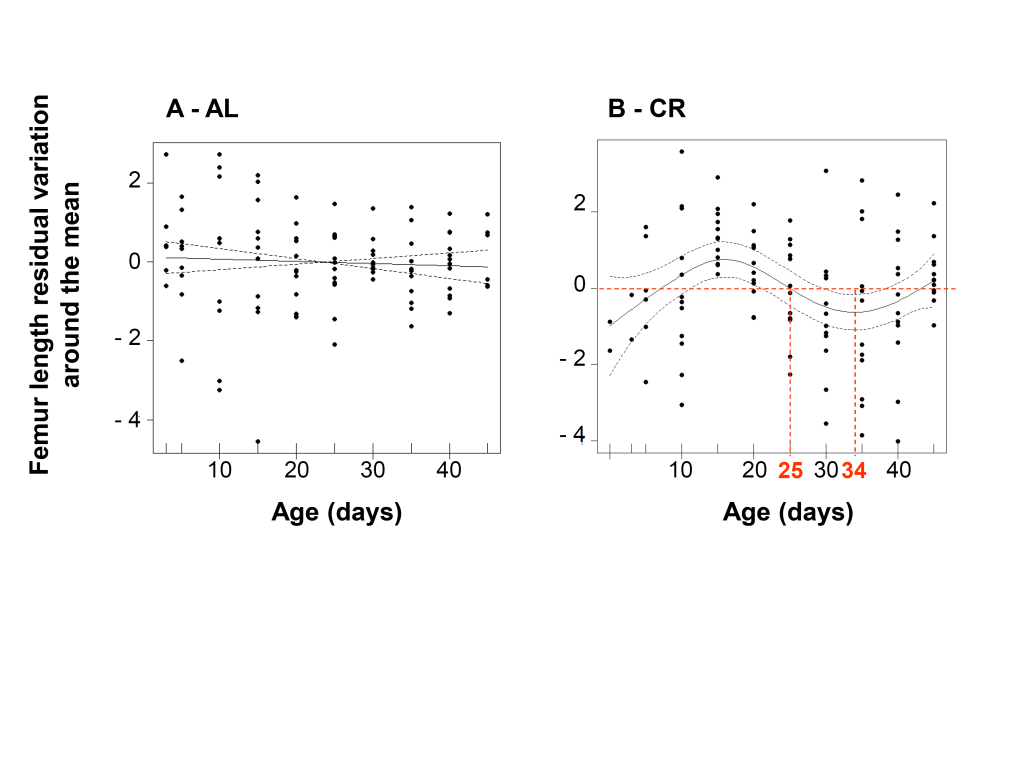

Supplement: Figure S3 — Effect of food availability to lactating mothers on the regularity of offspring femur growth. Temporal variation of femur length residuals (i.e., adjusted for Gompertz growth) of offspring from females fed ad libitum (AL, panel A.) and calorie restricted females (CR, panel B.). A value of 0 for the spline term indicates average residual femur length. The spline curve describing the smoothed effect of age (solid line) was estimated using a GAMM. Dashed lines depict 2 standard error point-wise confidence bands, and black dots provide partial residuals. In panel B., red dashed lines and bold labels delimit the period of delayed growth. (TIF) [file pone.0041477.s003.tif]
